# Environmental Molecular Effect on the Macroscale Friction Behaviors of Graphene

**DOI:** 10.3389/fchem.2021.679417

**Published:** 2021-06-23

**Authors:** Panpan Li, Bo Wang, Li Ji, Hongxuan Li, Lei Chen, Xiaohong Liu, Huidi Zhou, Jianmin Chen

**Affiliations:** ^1^ Key Laboratory of Science and Technology on Wear and Protection of Materials, Lanzhou Institute of Chemical Physics, Chinese Academy of Sciences, Lanzhou, China; ^2^ Center of Materials Science and Optoelectronics Engineering, University of Chinese Academy of Sciences, Beijing, China; ^3^ State Key Laboratory of Solid Lubrication, Lanzhou Institute of Chemical Physics, Chinese Academy of Sciences, Lanzhou, China

**Keywords:** graphite, graphene, macroscale tribology, sliding interfacial structure, atmosphere

## Abstract

This study investigated the friction behavior of graphene in air and nitrogen atmosphere environments. The microstructural evolution caused by the variation of atmosphere environments and its effect on the friction coefficient of the graphene is explored. It is demonstrated that graphene can exhibit excellent lubricating properties both in air and nitrogen atmosphere environments. In air, a highly ordered layer-by-layer slip structure can be formed at the sliding interface. Oxygen and H_2_O molecules can make edge dangling bonds and defects passive. Thus the interaction between the nanosheets and the layers of nanosheets is weak and the friction coefficient is low (0.06–0.07). While the friction coefficient increases to 0.14–0.15 in a nitrogen atmosphere due to the interaction of defects generated in the sliding process, the nitrogen molecules with lone pair electrons can only make the nanosheets passive to a certain degree, thus the ordered slip structure is destroyed and friction is higher. This work reveals the influence of environmental molecules on the macroscale tribological performances of graphene and its effect on the microstructure at the sliding interface, which could shed light on the lubricating performance of graphene in environmental atmospheres and help us to understand the tribological behaviors of graphite at the macroscale.

## Introduction

Reducing friction and wear losses has great value for the moving components of sliding conditions (Holmberg and Erdemir, 2017). As the typical layered material, graphite is used extensively as the solid lubricant due to the weak interaction of van der Waals force between layers (Geim and Novoselov, 2007). It is indicated that the tribological behaviors of graphite are easily affected by atmospheric environments, and it exhibits low friction in the air atmosphere and high friction in vacuum or inert atmospheres (Savage, 1948; Savage and Schaefer, 1956). During the last few decades, most research and practical applications have been concerned with the tribological performance of graphite (Lancaster and Pritchard, 1981) and few studies have focussed on the intrinsic microstructure that determines the tribological properties of graphite. On the one hand, the structural change of three-dimensional (3D) graphite is difficult to observe. On the other hand, the system of practical applications is usually complicated. Therefore, the exploration of the effect of the atmospheric environment on the intrinsic structure of the underlying origin of macroscale tribological performance is difficult and requires further investigation. Meanwhile, some lubricating mechanisms of graphite have been proposed to explain the performance of graphite in various conditions (Bollmann and Spreadborough, 1960; Bryant et al., 1964) but there are still disputes about the precise nature of these mechanisms.

Microscale tribological studies of lubricating materials, especially the studies of structure could guide understanding of the origin of the tribological properties and macroscale lubricating mechanism (Zheng and Liu, 2014; Zhang et al., 2019). As the basic unit of graphite, graphene possesses a typical two-dimensional (2D) thin-paper nanosheet structure that endows the graphene with excellent properties including fascinating physical, electronic, and chemical properties (Zhu et al., 2010; Avouris and Dimitrakopoulos, 2012; Badhulika et al., 2015). Graphene shows outstanding tribological properties (Berman et al., 2014; Berman et al., 2015) and has attracted considerable attention in the field of tribology. Macroscale tribological research of graphene started in recent years and is increasing gradually. The graphene coatings dropped on the steel substrate have the ability to reduce friction and wear in the air and dry a nitrogen atmosphere (Berman et al., 2013a; Berman et al., 2013b), but a lower friction coefficient is still required. Similar to other carbon materials, humidity, or atmosphere also has an obvious influence on the friction performance of graphene (Erdemir, 2001; Yen et al., 2004; Bhowmick et al., 2014). As a new emerging solid lubricant, thin-papered structure graphene possesses plenty of exposed surfaces that can provide convenience for the characterization of the microstructure of graphite, which could be accessible for exploration of the intrinsic structure of the sliding contact interface of graphene (Maciel et al., 2020). The macroscale friction coefficient of graphene is also dependent on its microstructure, so it can be presumed that graphene with intact nanosheets can show excellent lubricating performance. In particular, the highly ordered slip structure at the sliding interface lets graphene obtain macroscale superlow friction (Song et al., 2017), and ideal layered slipping microstructure is the prerequisite for graphene to exhibit outstanding macroscale tribological behaviors (Li et al., 2020; Gao et al., 2020). In recent years, research on the tribological properties of graphene are mainly focused on the microscale and in theory (Hirano and Shinjo, 1990) (Dienwiebel et al., 2004; Song et al., 2018) and can provide guidance for macroscale research. The microscale friction occurs at the ideal small contact interface, and the intact nanosheet of graphene can get low friction (Hod et al., 2018; Chen and Li, 2020). However, at the macroscale, the contact interface has large and plentiful factors that influence the frictional performance (Cooper et al., 2019; Yu et al., 2020). In particular, graphene nanosheets have a high specific surface area and more easily absorb surrounding gas molecules when exposed to an atmosphere, which would easily affect macroscale tribological properties. Previous macroscale studies indicate that graphene exhibits low friction in moisture, hydrogen, or air environments with active gas molecules and show the response to the surrounding atmosphere environments (Berman et al., 2015; Li et al., 2017; Gao et al., 2018). To understand the underlying origin of the tribological performance of graphene, the relationship between the changes of microstructure and the tribological properties still needs to be explored and could shed light on the macroscale lubricating mechanism of graphene (mechanically exfoliated graphene was used in this work), thus to help understand the macroscale lubrication mechanism of graphite.

Based on this, the macroscale tribological properties of graphene in air and nitrogen atmospheres were explored, and the response behaviors to the surrounding atmospheres were studied. Then the corresponding microstructure of the sliding interface and the morphologies of the frictional pair were characterized by the transmission electron microscope (TEM) and Raman spectra as well as optical image to investigate the influence of atmosphere environments on the microstructure and the effect on the macroscale frictional behavior of graphene. The research of the structural evolution induced by the surrounding atmospheres and their influence on the friction coefficient during the atmosphere change helps reveal the macroscale tribological performance of graphene and thus furthers understanding of the lubricating mechanism of graphite at the macroscale, which could be significant for providing guidance for graphene (graphite) to be used as a solid lubricant at the macroscale.

## Methods

### Materials and Sample Preparing

**FIGURE 1:**
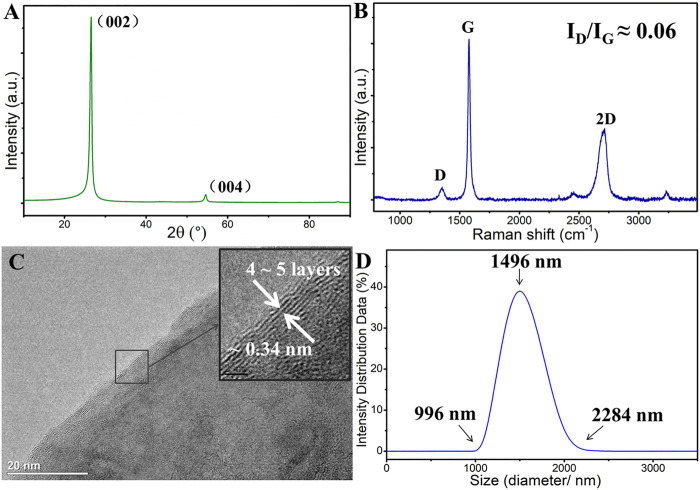
Original microstructure of graphene: **(A)** XRD patterns, **(B)** Raman spectroscopy, **(C)** HRTEM morphology of graphene, **(D)** Lateral size of graphene nanosheets.

**FIGURE 2:**
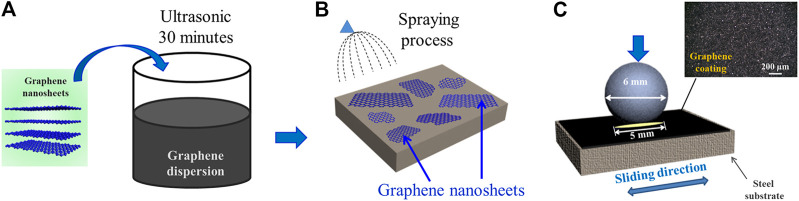
**(A)** Schematic of preparation of graphene dispersion, **(B)** schematic of preparation process of graphene coating, **(C)** schematic of friction test.

Graphene powder was purchased from Nanjing Jicang Nano Technology Company Limited (Nanjing, China), and the graphene nanosheets are prepared by mechanical exfoliation. Analytical grade volatile alcohol (Tianjin, China) was commercially obtained. The purchased graphene powder and volatile alcohol without any chemical treatment were chosen to explore the inherent properties of pure graphene. The original structure of graphene is shown in Figure 1. The graphene powder above was dispersed in 100 ml volatile alcohol at a mass concentration of 2 g/L, and then the dispersion was sprayed on the M2 steel substrate immediately after 30 min ultrasonic (Figure 2A shows the sample preparation process). Before the spraying process, the M2 steel substrate was sanded by 1,000 grit sandpaper. During the spraying process, the dry high-purity nitrogen was used as the carrier gas at a pressure of 0.2 MPa (the schematic was shown in Figure 2B), and the obtained samples were dried in a vacuum at 80°C for 1 h to afford the graphene coatings.

### Frictional Test

A pin-on-disk CSM tribometer (CSM, Switzerland) was used to conduct the frictional tests of the graphene coating. The counterpart ball commercially purchased AISI52100 steel ball (Diameter: Ø = 6 mm, Roughness: Ra∼20 nm) was chosen. The friction experiments were performed at room temperature (23 ± 2°C), and the atmosphere is controlled by the chamber that the tribometer carried itself (Figure 2C is the schematic for the assembly of the sliding pair). Before the friction test under nitrogen atmosphere, dry nitrogen gas was poured into the chamber for about 10 min to eliminate the air atmosphere that may exist in the chamber, then the friction test start (relative humidity ≤ 2%), namely, the entire graphene is exposed to the nitrogen atmosphere at this time. When performing the friction test in the air atmosphere, the chamber of the friction machine was opened and the sample was exposed to air, and then the friction test start. The friction tests were performed in reciprocating mode, and the test frequency was 6.37 Hz (maximum sliding speed was 10 cm/s), the full amplitude was 5 mm, and maximum Hertz contact pressure is 0.5 GPa calculated by the Hertz contact theory (equivalent to the applied load was 0.5 N), respectively. The samples chosen for the structural characterization were obtained from the stable friction stage when the material has not failed. Before each friction experiment, the tribometer was calibrated automatically to eliminate accidental errors.

### Characterization

The phase composition of the original graphene powder was identified by X-ray diffraction (XRD, Bruker, D8Discover25, Germany), and the operating voltage was 40 KV and the operating current was 40 mA, and copper target (λ ≈ 0.154 nm) was used. The Raman spectra of graphene surface, wear tracks, and wear scars of the counterpart ball were analyzed by Raman spectroscopy (Renishaw, Raman inVia, England) using a wavelength of 532 nm (2.3 eV) lase through the 50X objective lens. The optical images of the original surface, wear scars, and wear tracks of graphene coating were investigated by optical microscope (Olympus, STM6, Japan). The graphene powder was dispersed in ethanol and ultrasonic for approximately 30 min, and then the dispersions were transferred to the Cu grid to observe the morphologies and structures. The structural evolution of the sliding interfaces after 30 min rubbing was transferred to the Cu grid to observe their structures and morphologies. The morphology of graphene powder and sliding interface was characterized by Transmission Electron Microscopy (TEM, FEI Tecnai G2, TF20, United States) and high resolution transmission electron microscopy (HRTEM). The lateral size of the graphene nanosheets was tested by the Zetasizer Nano ZS laser dynamic scattering instrument (ZEN 3600, United Kingdom).

## Results

### Different Friction Coefficient of Graphene

Figure 1 shows the original structure of graphene. The XRD pattern of graphene shows that there is an evident diffraction peak at∼25°corresponding to the (002) crystal base plane of graphene (Figure 1A). Figure 1B is the Raman spectroscopy of the original surface of graphene coating, and the low D peak intensity indicates that graphene nanosheets possess few defects or functional groups (the ratio of I_D_/I_G_ is about 0.06). The graphene nanosheet is about 4–5 layers, and *d*-spacing is about 0.34 nm (Figure 1C), indicating that graphene nanosheets are typical 2D lamella structures.

The size of the graphene nanosheets is shown in Figure 1D. It can be seen that the lateral size graphene sheets are relatively uniform, with a distribution range of 1–2.3 µm. Figure 2 shows the preparation process of graphene dispersion (Figure 2A) and graphene coating (Figure 2B). Figure 2C is the schematic of the reciprocating friction test, and the inset shows the surface morphology of the original graphene coating, which covers the surface of the whole substrate. All process does not introduce any pollutants and interferences, thus the intrinsic tribological properties of graphene are studied. In addition, the tribological performance of graphene is evaluated by a pin-on-disk tribometer in reciprocating mode, and the CSM friction testing machine has a chamber that can control the atmospheric environments.

**FIGURE 3:**
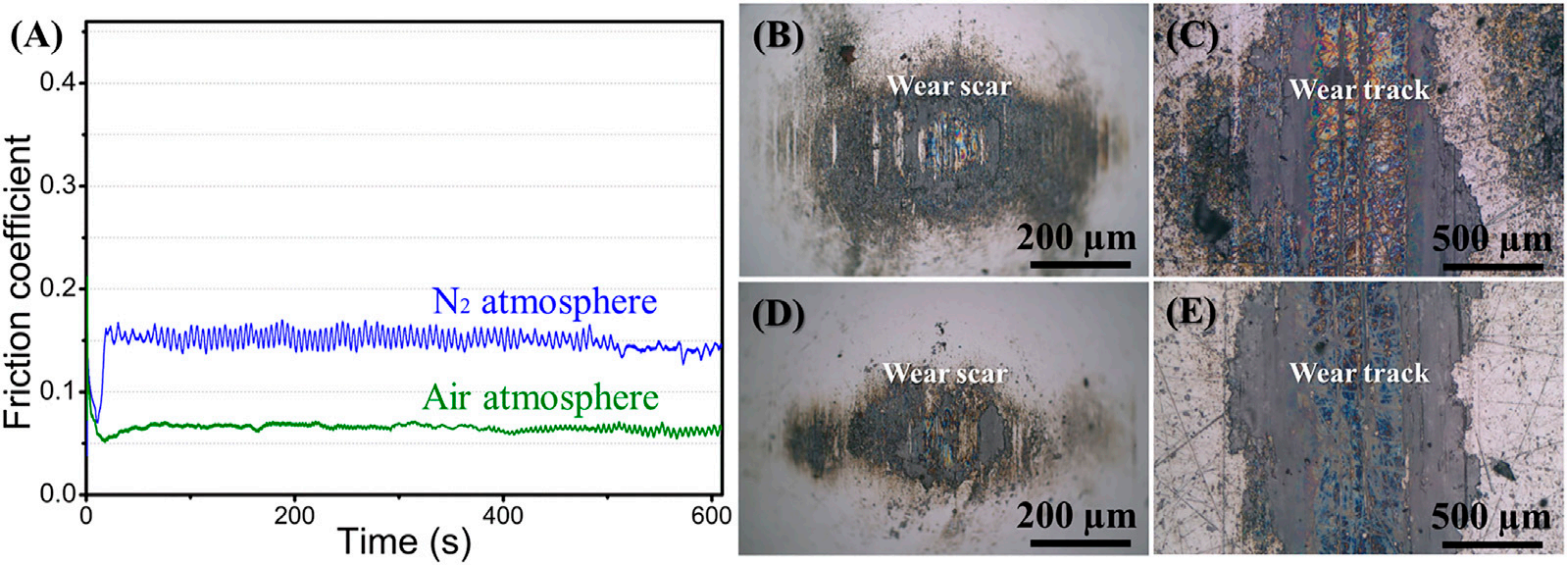
**(A)** Curves of friction coefficients of graphene coatings in different atmospheres, optical images of **(B)** wear scar in nitrogen, **(C)** wear track in nitrogen, **(D)** wear scar in air, **(E)** wear track in the air.

The friction coefficient curves of graphene in air and nitrogen atmospheres are shown in Figure 3A. The friction coefficient can be quickly stabilized, and the friction coefficient of graphene in air (about 0.06–0.07) is significantly lower than that in nitrogen (about 0.14–0.15). The friction coefficient in nitrogen shows fluctuations, while the friction coefficient in air is relatively stable. The optical images of the wear scars and the wear tracks after 10 min sliding process in air and nitrogen atmospheres are given in Figures 3B–E. Both uniformly transferred film and lubricating film can be formed on the tribo-pair, whether in the air or in a nitrogen environment. The graphene coating plays an effective lubricating role and exhibits relatively low friction. Namely, in the macroscale friction test, when the good transferred film and lubricating film are formed on the tribox-pair (Kohlhauser et al., 2020), graphene coating can reduce friction effectively.

### Responsiveness of Graphene to Atmospheres Environment

**FIGURE 4:**
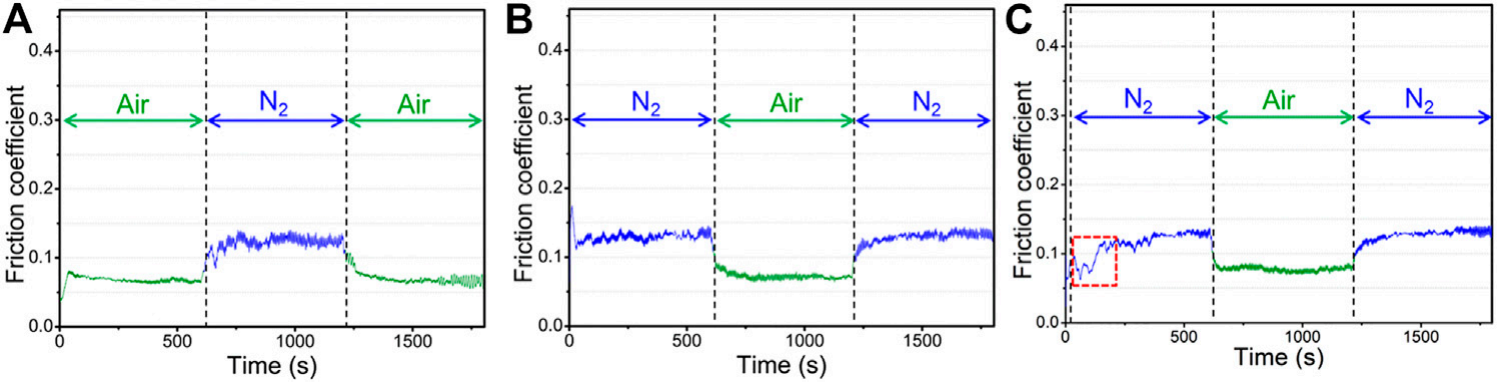
Variation of the friction coefficient of graphene coatings in alternated atmospheric process: (**A**) the friction test process that 10 min in air, and 10 min in nitrogen then 10 min in air again, **(B)** the friction test process that 10 min in nitrogen, and 10 min in air then 10 min in nitrogen again, **(C)** a short-term running-in test and subsequent formal friction test process that 10 min in nitrogen, and 10 min in air then 10 min in nitrogen again.

In order to further explore the variation of friction coefficient of graphene caused by the atmosphere environment, and to reduce the influence of the system error of the instrument itself during the friction experiment, two processes for creating atmospheric environment changes were introduced under otherwise identical conditions except for the initial environmental atmosphere. One of which was the process from air to nitrogen and then to air again (Figure 4A) and the other is from nitrogen to air and then to nitrogen again (Figure 4B). In these processes, the friction tests were carried out continuously, and only the environmental atmosphere was variable. Figures 4A,B shows the variation of the friction coefficient of graphene during the variable process of atmosphere. It is indicated that graphene exhibits a sensitive response to atmospheres. A friction coefficient of 0.13–0.15 was measured in a nitrogen atmosphere, which is higher than that of 0.07 in air and is nearly the same as the above friction coefficient of graphene in different atmospheres (about 0.06–0.07 in air and 0.14–0.15 in nitrogen). The two processes both show that there is a stage that the friction coefficient gradually rises when the friction test from air enters nitrogen. After a period of time, the value of the friction coefficient stabilizes at that of the nitrogen (0.13–0.15). However, there is not the same gradual change process when the friction test from nitrogen enters air. In addition, the friction coefficient of graphene during the two cycles can be returned to the same degree as the friction coefficient in the pure environmental atmosphere, which also indicates that there are obvious responsive behaviors of graphene to the external environmental atmosphere. Figure 4C shows the friction coefficient curve of the graphene coating that a short-term running-in test was introduced to the air before the subsequent formal sliding test. Different from the friction test, a test was also conducted in a nitrogen atmosphere. After the short-term running-in test in the air, there is a significant fluctuation stage in the early stage when the friction test enters the nitrogen (the red box in Figure 4C). At this time, the friction coefficient is low and almost close to the friction coefficient in the air; a few minutes later, the friction coefficient increases gradually and finally approaches 0.14–0.15 that of the value in nitrogen. The stage that friction coefficient slowly rises also exists in the next friction process that the atmosphere from the air enters the nitrogen.

Graphene coatings show excellent macroscale tribological properties whether in nitrogen or an air environmental atmosphere. The friction coefficient in the air is lower than that in nitrogen. A short-term running-in test in the air helps graphene to obtain low friction, especially in the initial stage of the friction test when the atmosphere from air enters the nitrogen. The friction coefficient of graphene shows an obvious response to the atmospheric environments, and the variation of the external atmospheres has a significant impact on the tribological performances of graphene.

### Variation of Friction Coefficient and Corresponding Microstructure

**FIGURE 5:**
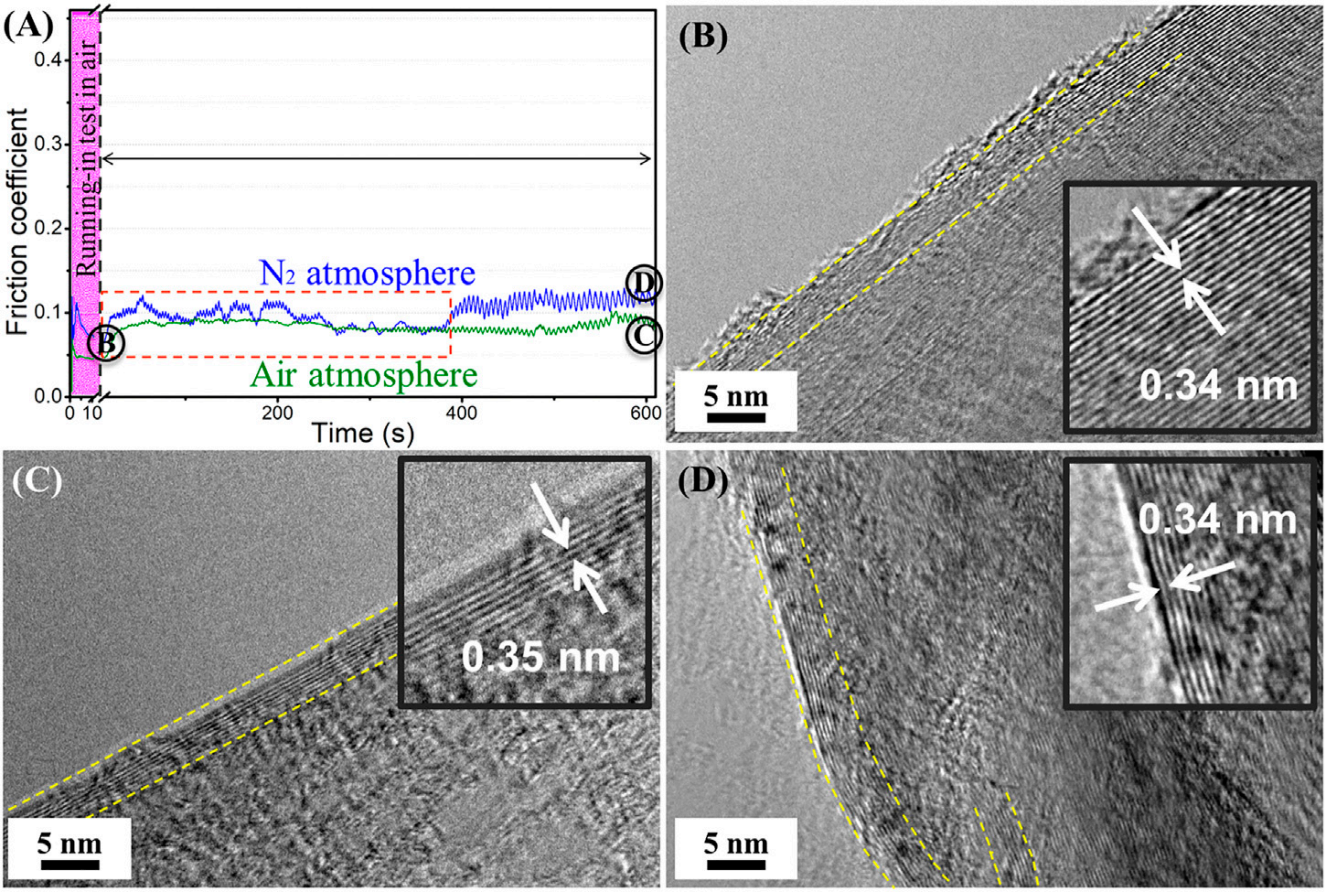
The friction coefficient and the corresponding microstructure of the sliding interface of graphene: **(A)** friction coefficient curves of graphene in air and nitrogen after the short-term running-in test, (**B)** sliding interface structure of graphene after the short-term running-in test (point B), **(C)** sliding interface structure of graphene of formal friction test in air (point C), **(D)** sliding interface structure of graphene of formal friction test in nitrogen (point D).

Figure 5A shows the friction coefficient curves of graphene of the formal sliding test in air and nitrogen atmospheres after the short-term running-in test. It can be seen that the friction coefficient shows a downward tendency in the process of the short-term running-in test period. Then the friction coefficient fluctuates in the subsequent formal sliding test in nitrogen, and the friction coefficient value is less than that of the friction test performed in the nitrogen environment directly. After a period of time, the friction coefficient becomes stable and gradually approaches the value that of in nitrogen. In the subsequent sliding test in air after the short-term running-in test, the value of the friction coefficient is almost equal to the value of the friction test directly in air, but the friction coefficient is more stable. The friction coefficient fluctuates and the degree of fluctuation is close to that in the air. Even a very short-term running-in test in the air before the formal friction experiment can improve the macroscale tribological properties of graphene. The microstructure of the sliding interface of the running-in test process is shown in Figure 5B. The HRTEM morphology (Point B in Figure 5A) shows that a highly ordered layer-by-layer sliding structure is formed at the sliding contact interface, and the number of layers is about 35, while the original graphene nanosheets are about 4–5 layers, which also indicate that the slip occurs between the nanosheets of graphene (Song et al., 2017). The distance between graphene nanosheets is larger than the layers of graphene nanosheets, so the interactions between the nanosheets are weaker and allow graphene to exhibit a low friction coefficient at the macroscale. Figure 5C,D show the sliding interface structure of graphene of the formal friction tests in air and nitrogen, respectively. Results indicate that the flat ordered slip structure still exists on the sliding contact interface after 10 min friction test in air, and the number of the slip structure is about 8–10 layers, meanwhile the *d*-spacing of the layers of the graphene nanosheets is about 0.35 nm. Nevertheless, the flat ordered slip structure becomes bent after 10 min friction test in nitrogen, and the number of the slip structure is also about 8–10 layers. The slip still occurs between the graphene nanosheets, and *d*-spacing is about 0.34 nm, slightly less than the *d*-spacing after the friction test in air. In an air atmosphere, the nitrogen molecules have a diameter of approximately 0.42 nm and the oxygen molecules have a diameter of approximately 0.38 nm H_2_O molecules have a diameter of about 0.32 nm and a certain chemical activity exists. There are only nitrogen molecules in the nitrogen atmosphere, so the H_2_O molecules may enter into the layers of graphene nanosheets, inducing the *d*-spacing of layers to increase slightly. The larger *d*-spacing is advantageous for graphene, enabling a low friction (Liu et al., 2019; Rosenkranz et al., 2020). This may be a part of the reason that the friction coefficient of graphene in the air is lower than in nitrogen. In addition, the oxygen and H_2_O molecules in the air can cause the edge dangling bonds and defects of nanosheets to become passive (Bhowmick et al., 2015), meaning the interaction between layers of graphene nanosheets is weak. These two aspects mean that the friction in air is significantly lower than that in nitrogen. In the macroscale friction test, the atmospheric molecules have an obvious impact on the microstructure of graphene, even a short-term running-in process can be induced in the formation of the ordered layered slip structure, and the formation of the ordered slip structure provides a prerequisite of low friction coefficient that is of benefit for graphene in exhibiting the excellent macroscale tribological performances (Figure 5A). The ideal flat ordered slipping structure enables the subsequent friction test in air (Figure 5C), and the friction coefficient keeps a relatively low state (0.05–0.06), although it does fluctuate to a certain extent. However, the flat ordered slipping structure is damaged in the subsequent friction test in nitrogen and the nanosheets become bent (Figure 5D), which could because the nitrogen molecules cannot insert the interlayer of graphene nanosheets to make the defects passive, causing the interaction of the layers, meaning a high friction coefficient (0.14–0.15) is obtained.

**FIGURE 6:**
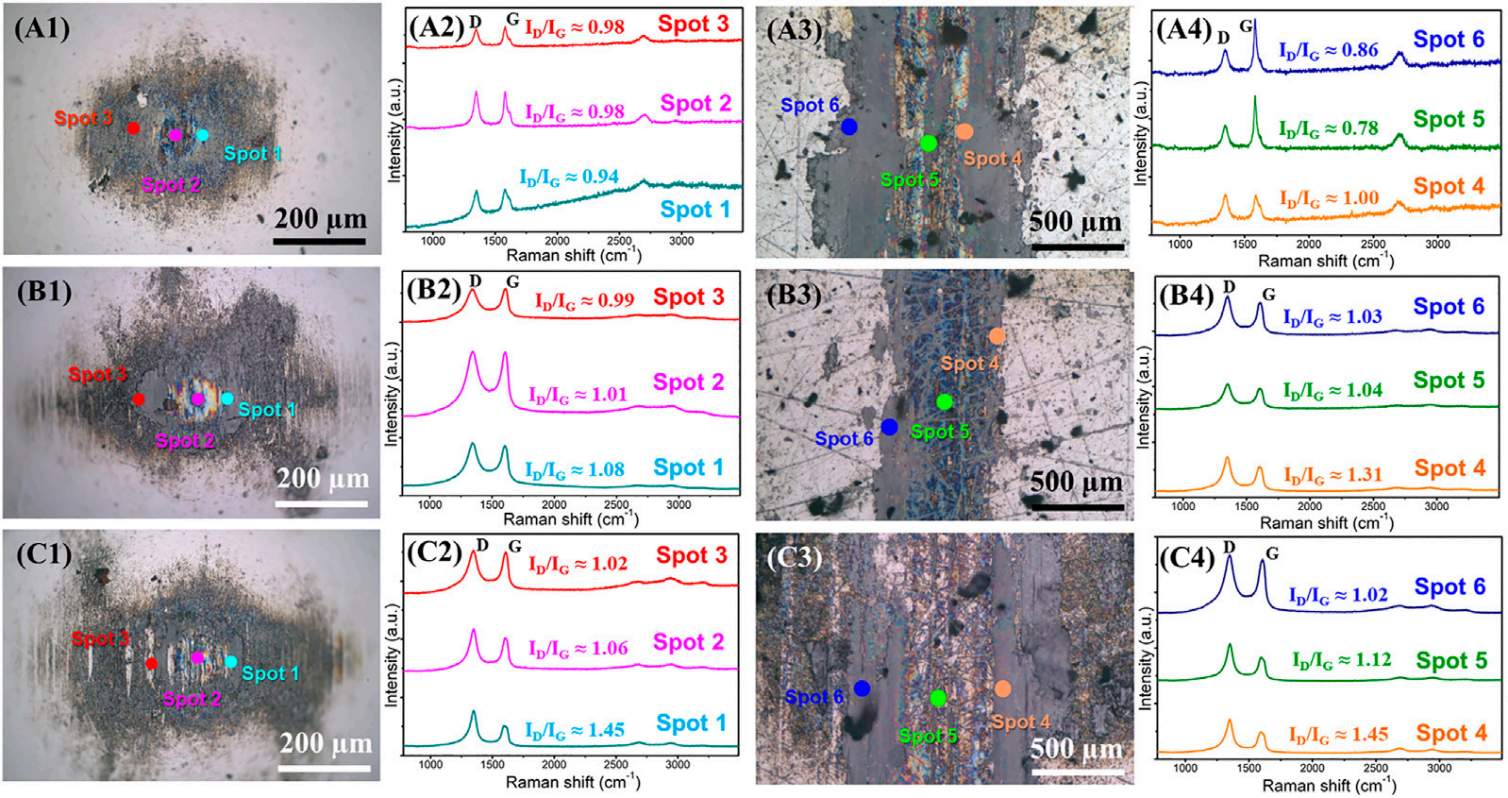
Morphologies of the tribox-pair and the Raman spectra of graphene during the variable process of atmosphere environment: (**A1, A2, A3, A4**) the optical images of the wear scar and wear track after the short-term running-in test and the corresponding Raman spectra at labeled spots in the optical images, (**B1, B2, B3, B4**) the optical images of the wear scar and wear track of the subsequent friction test carried out in air for 10 min and the corresponding Raman spectra at labeled spots in the optical images, **(C1, C2, C3, C4)** the optical images of the wear scar and wear track of the subsequent friction test carried out in nitrogen for 10 min and the corresponding Raman spectra at labeled spots in the optical images.

The surface morphologies of wear scars and wear tracks and the Raman spectra during the variable process of atmospheres environment are shown in Figure 6. The three stages (corresponding to Figure 5) are included, a short-term running-in test in air, 10 min friction test in air after the running-in test, and 10 min friction test in nitrogen after the running-in test. It is indicated that even after a short time running-in test, the uniformly transferred film, and lubricating film can form on the friction pair (Figures 6A1, A3). Raman spectra show that three obvious peaks, including D peaks, G peaks, and 2D peaks, and at the location of the transferred film and the lubricating film graphene coating exists (Ferrari et al., 2006). After a short-term running-in test, the intensity of the D peak increases compared with the original graphene (Figure 1B), the ratio of I_D_/I_G_ reach about 0.78–0.98 from about 0.06 of original nanosheets. However, the HRTEM morphology shows (Figure 5B) that the microstructure of the sliding contact interface becomes ordered, though the friction process destroys the graphene nanosheets. Spots 1, 2, and 3 in Figure 6A1 represent the transferred film in the contact state near the center, the transferred film in the contact state at the center, and the thicker nanosheets far from the center, respectively. Spots 4, 5, and 6 in Figure 6A3 represent the lubricating film in the contact state near the center, the lubricating film in the contact state at the center, and the thicker nanosheets far from the center, respectively (the other spots below represent the same meaning). After the same time (10 min) friction test in different atmospheres (air, nitrogen), the ratio of I_D_/I_G_ increases further. Nevertheless, the microstructure of the sliding interface is ordered in air (Figure 5C), although the friction process further damages the structure of the graphene nanosheets. Because oxygen and H_2_O molecules in air can passivate the defects and edge dangling bonds (Li et al., 2017), the interaction between the graphene layers is weak and the friction coefficient is low. After 10 min friction test in nitrogen, the intensity of the D peak increases and is higher than that in the air. HRTEM topography (Figure 5D) shows that the graphene nanosheets of the sliding interface become bent, which indicates that the structure of graphene is chaotic and disordered. The nitrogen molecule is inert, and its lone pair electrons possess the conjugation effect with the π bond of the graphene carbon layers (Ji et al., 2009), which also plays a role in the passivation to a certain degree. Moreover, at the initial stage that the friction test enters into air from the nitrogen atmosphere, the H_2_O molecules continue to interact with the high-energy defects strongly, and the nitrogen molecules only can enter into the graphene nanosheets and not the layers of graphene nanosheets. The H_2_O molecules play a role in passivation and weaken the interaction of the layers of the graphene nanosheets, thus the friction coefficient remains low. With the friction test progressing, more defects are exposed and then the layers begin to interact with each other, causing the friction to fluctuate and increase, though nitrogen molecules can make the edge dangling bonds of the nanosheets passive through the p-π conjugation effect. Finally, the friction coefficient reaches the level of 0.14–0.15 in nitrogen. The HRTEM morphology also indicates that the flat ordered slip structure of the graphene laminates was destroyed and bent. Thus, the interaction between graphene layers becomes stronger, and the friction coefficient is higher than that in air.

Raman spectra and the morphologies of the sliding interface demonstrate that the structure of graphene nanosheets is inevitably destroyed during the friction process, and the ordered slip structure is formed at the sliding interface, which is the structural prerequisite for graphene to obtain macroscale low friction. The strength of the interaction of the formed slip structure determines the friction coefficient of graphene at the macroscale. The active oxygen and H_2_O molecules in the air atmosphere can passivate edge dangling bonds and defects. Thus, the interaction between layers is weak, and the flat ordered slip structure forms at the sliding contact interface, and the friction coefficient is low. While in a nitrogen atmosphere, nitrogen molecules also can passivate edge dangling bonds to a certain degree, due to the conjugation effect between its lone pair electron and the *π* bonds of the graphene carbon, thus letting graphene exhibit the effectively lubricating performance. But there is still interaction between the layers of graphene nanosheets, so the friction coefficient is higher than that in air.

### The Evolution of Microstructural and Tribological Performance

**FIGURE 7:**
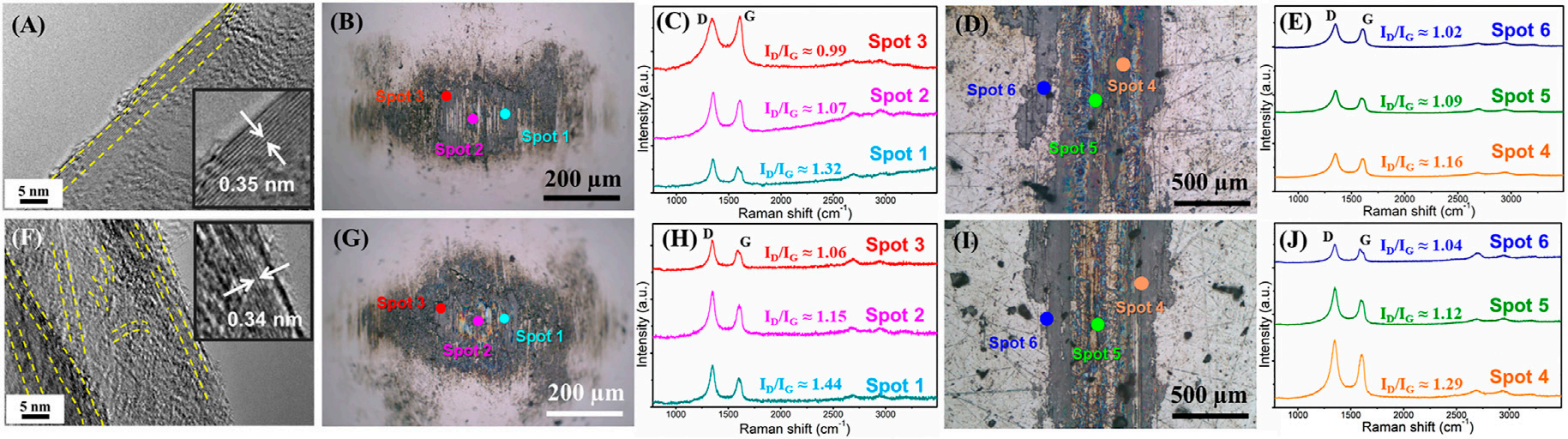
Morphologies and the microstructure of the friction interface of graphene during the subsequent variable process of atmosphere environment. The friction test in air after the friction test in nitrogen atmosphere (first time 10 min friction test in air in 4C): **(A)** HRTEM morphology of the sliding interface, **(B)** optical images of the wear scar, **(C)** Raman spectra of labeled spots in the optical images of the wear scar, **(D)** optical images of the wear track, **(E)** Raman spectra of labeled spots in the optical images of the wear track, the next friction test in nitrogen after the friction test in air atmosphere (second time 10 min friction test in nitrogen in 4C), **(F)** HRTEM morphology of the sliding interface, **(G)** optical images of the wear scar, **(H)** Raman spectra of labeled spots in the optical images of the wear scar, **(I)** optical images of the wear track, **(J)** Raman spectra of labeled spots in the optical images of the wear track.

To investigate the microstructural evolution of the sliding contact interface caused by the variation of atmosphere and its effect on the friction coefficient of the graphene in the entire process, we further explored the microstructure in subsequent tests. This examined an atmosphere from nitrogen to air and we conducted a subsequent friction test in a nitrogen environment (Figure 4C). Figure 7 shows the morphologies and the Raman spectra of a friction test in air (first time 10 min friction test in air in Figure 4C) and nitrogen (second time 10 min friction test in nitrogen in Figure 4C). It can be seen that a flat and ordered slip structure (Figure 7A) forms when the friction test enters air, while the flat and ordered slip structure was destroyed and became bent (Figure 7F) when the friction test enters the nitrogen a second time. At this time, the friction test was carried out for 30 min, and the graphene nanosheets at the sliding contact interface became damaged and bent. The *d-*spacing of the layers of the sliding structure is 0.34 nm, slightly smaller than that in air (0.35 nm). The uniform transferred films (Figures 7B,G) and lubricating films (Figures 7D,H) still form on the friction pai at this time, which is also similar to the results above, and graphene can obtain low friction coefficients as well (0.06–0.07 in air and 0.14–0.15 in nitrogen). Three obvious peaks, including D peaks, G peaks, and 2D peaks are shown in Raman spectra where the transferred film or the lubricating film exists. The intensity of the D peak increases, which further illustrates that the process of friction destroys intact graphene nanosheets. The chosen standard of spots 1, 2, 3, 4, 5, and 6, as seen in Figure 7 are the same as those described in Figure 6. During the friction test in air, the intensity of the D peak increases further and the graphene nanosheets became more disordered, but the friction coefficient is in the low state (0.06–0.07), which is due to the passivation of the oxygen and H_2_O molecules of air. The interaction between the layers of graphene nanosheets is weak, and the sliding resistance between layers of graphene nanosheets is small. In subsequent friction test in nitrogen, the defects that formed during the friction process interact with each other, and the graphene nanosheets began to be damaged and become bent and ordered, causing the intensity of the D peak to increase further. The friction coefficient also shows an upward trend and fluctuates in the final stage.

The tribological properties and structural evolutions of graphene during the whole variation process of the atmosphere environments were studied, including a short-term running-in test in air, 10 min friction test in nitrogen, then a 10 min friction test in air, and a 10 min friction test in nitrogen again. During the whole process, the graphene coatings were not worn out and failed, and the intrinsic microstructure of graphene was utilized to explore the macroscale tribological behaviors of graphene.

## Discussion

**FIGURE 8:**
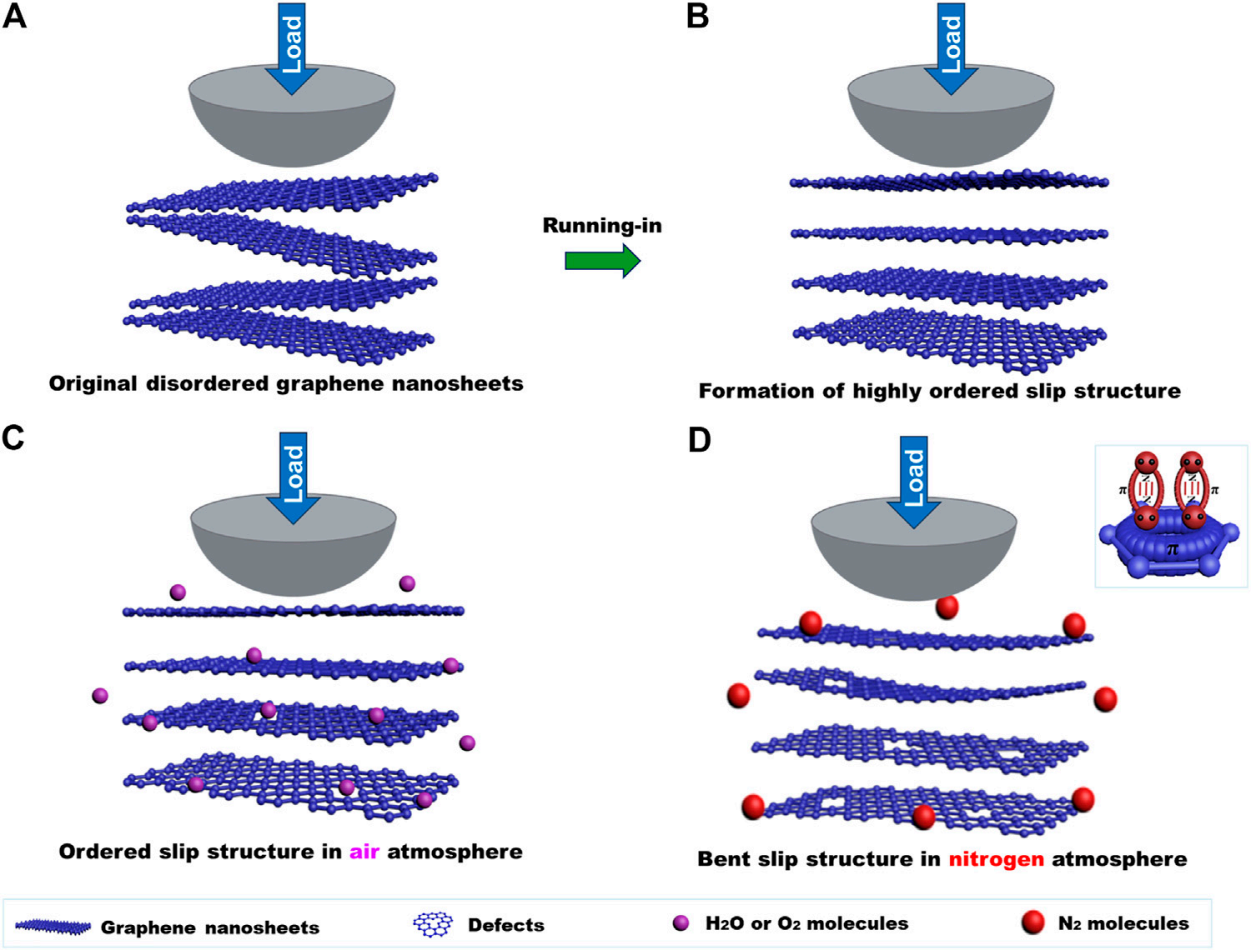
Macroscale lubricating mechanism of graphene in an atmospheric environment: **(A)** the original disordered graphene nanosheets of the graphene coating, **(B)** the ordered slip structure formed after a short time in the air atmosphere, **(C)** the flat ordered layer-by-layer slip structure in the air after friction, **(D)** the bent graphene nanosheets after friction in nitrogen.

Graphene shows a sensitive response to atmospheres in the macroscale friction test process, and its friction coefficient in air is lower than that of nitrogen. The uniform transferred film and lubricating film mean that the graphene coating exhibits low friction during the friction process. The optical images, Raman spectra, and SEM morphology show that the transferred film and lubricating film are graphene nanosheets. Therefore, the difference in the friction coefficient of the graphene coating is mainly determined by the microstructure of the graphene at the sliding contact interface. The highly flat ordered layer-by-layer slip structure at the sliding interface can be formed in a short time in air (Figures 8A,B), and the formed layer-by-layer slip structure is the structural prerequisites for graphene to obtain the low friction coefficient (Song et al., 2017; Li et al., 2020). At the macroscale, the slip occurs between the nanosheets of graphene, and the intrinsic friction is dependent on the interaction between its nanosheets and the layers of graphene nanosheets. When the interlayer interaction is weak, graphene can obtain a low friction coefficient (Hirano and Shinjo, 1990; Hod et al., 2018). However, the graphene nanosheets are inevitably destroyed during the friction process, and the Raman spectroscopy also illustrates that after friction test in an air or nitrogen atmosphere, the intensity of the D peak increase indicates that defects of graphene nanosheets increase. The active oxygen and H_2_O molecules in the air can passivate edge dangling bonds and defects of graphene, thus the interaction between graphene nanosheets is weak; especially the diameter of H_2_O molecule, which is about 0.32 nm, which may enter the interlayer of the graphene nanosheets and pacify the defects that formed during the friction process. Therefore, the interaction between the nanosheets and the layers of nasheets is weak (Figure 8C) and a flat ordered layer-by-layer slip structure can form at the sliding interface though the friction process, causing damage to the graphene nanosheets. Therefore, the friction coefficient lowers to 0.06–0.07. The nitrogen molecule has lone pair electrons that can share the electron cloud with the *π* orbital of graphene and possess the p-π conjugation effect, thus making the edges dangling bonds of graphene nanosheets passive, and weakening the interaction of graphene nanosheets to a certain degree. However, the diameter of the nitrogen molecules is larger than the *d*-spacing of graphene and it is generally difficult to enter the graphene layers. The defects generated during the friction process may interact with each other, and graphene nanosheets become bent when the friction test is conducted in nitrogen (Figure 8D). At this time, the graphene coating can still play a lubricating role, but the friction coefficient is higher than that in air, about 0.14–0.15.

The work above shows that gas molecules in different atmospheres have different effects on the microstructure of the sliding contact interface of the graphene during the friction process so that graphene exhibits different friction behaviors. The friction coefficient of graphene depends on the interaction between its layers and the nanosheets. Active gas molecules (such as oxygen and H_2_O molecules) can passivate the edge dangling bonds of graphene and the defects generated during the friction process. Thus, the interaction between the interlayer and the lamellae is weak and the friction coefficient is low. Inert gas molecules (such as nitrogen) possess the lone pair electrons that could interact with the *π* orbital of graphene and play a role in passivation to a certain degree, rendering the graphene to exhibit excellent macroscale lubricating properties.

## Conclusion

Graphene shows excellent lubricating behaviors in air and nitrogen atmosphere environments, which are attributed to the formation of the uniformly transferred film and lubricating film. The friction coefficient of graphene in air is about 0.06–0.07, which is lower than that of nitrogen (0.14–0.15). In particular, the active gas molecules in the air can passivate the defects and edge dangling bonds, and H_2_O molecules may enter the interlayer of the graphene nanosheets and passify the defects. The interaction between the graphene nanosheets and layers of graphene nanosheets is weak and the friction coefficient is low. The edge dangling bonds can also be passivated in nitrogen, due to the formation of conjugation between the lone pair electrons of the nitrogen molecules and the delocalized *π* bonds of graphene. It has been shown that the interaction of graphene nanosheets and layers of graphene nanosheets determine the macroscale friction coefficient of graphene. The active gas molecules in air can pacify the defects and dangling bonds of graphene nanosheets thus creating a low state of friction. The nitrogen molecules cause the interaction of graphene nanosheets to become passive through conjugation interaction. This work explores the influence of the molecular environment on the tribological behaviors of graphene, which could help understand the tribological performance of graphite in different environments.

## Data Availability

The original contributions presented in the study are included in the article/supplementary material, further inquiries can be directed to the corresponding authors.
